# Evaluating a ‘UBI Plus’ Intervention: A Needs-based Analysis of WorkFREE

**DOI:** 10.1007/s11205-026-03806-y

**Published:** 2026-03-10

**Authors:** Joel Lazarus, Sarath Davala, Maria Franchi, Neil Howard, Nick Langridge, Santosh Malviya, Vibhor Mathur

**Affiliations:** 1 Department of Social and Policy Sciences, 3 East, University of Bath, Bath, BA2 7AY UK; 2 India Network for Basic Income, Securanderabad, Hyderabad, Telangana 500015 India

**Keywords:** Needs, Universal Basic Income, Project Evaluation

## Abstract

As pilots in Universal Basic Income (UBI) pilots proliferate, there is increasing recognition that cash alone may not be enough to support envisioned transformation. Consequently, recent years have seen pilots in ‘UBI Plus’ - combinations of unconditional cash transfers with other social interventions. In this article, we present a mixed-methods needs-based evaluation of WorkFREE, a major UBI Plus pilot. Between 2020 and 2024, WorkFREE brought together UK and Indian researchers, a local NGO, and over 1,400 slum residents (295 households) in city name, India. WorkFREE participants received monthly unconditional cash transfers for eighteen months whilst participating in regular needs-focused ‘Plus meetings’. This article’s authors oversaw the design, implementation, and evaluation of WorkFREE both as a research project and pilot intervention. Locating human needs at an essential layer of existence and experience, we conducted all stages and aspects of our work using a needs-based approach. In this article, we use Manfred Max-Neef’s (1991) framework of fundamental human needs to evaluate WorkFREE’s UBI Plus pilot through a combination of quantitative data from three household surveys conducted over eighteen months and qualitative data conducted with participants over two years. We find compelling evidence that points to the synergic power of UBI Plus in supporting participants to more effectively and extensively meet not just their material, but also their psychological and relational needs. We recognise limits to our model and implementation - particularly with regard to responding to social, especially gendered, difference - but see enough evidence to advocate for UBI Plus and for needs-based approaches to research and social interventions.

## Introduction

Recent years have seen a significant increase across the world in public interest in, academic research into, and policy advocacy for the introduction of a Universal Basic Income (UBI). Here, we follow the Basic Income Earth Network’s (BIEN) definition of UBI, or just ‘basic income’, as ‘a periodic cash payment unconditionally delivered to all on an individual basis, without means-test or work requirement’.^1^ In this context, there has been a growing acknowledgement that cash transfers alone may be insufficient to support individual, collective, or societal change and researchers and activists have begun experimenting with ‘UBI Plus’ - the combination of unconditional cash transfers with other social interventions (Roelen et al., 2017; Howard et al., 2024; Mathur et al., 2025).

It is in this context that the authors of this article contributed to the design, implementation, and evaluation of WorkFREE - a major multi-year UBI pilot and research project that brought together researchers from the UK (University of Bath) and India (India Network for Basic Income) and an NGO and over 1,400 people living in urban informal settlements in city name, India to trial a UBI Plus combination of unconditional cash transfers and an unconditional relational and needs-centred approach to community organising.

Ours is a needs-based theory and practice (praxis). As we explain below, we designed WorkFREE according to a needs-based Theory of Change; implemented it through the integration of various needs-centred practical frameworks; and are now also evaluating it using a needs-based framework. The reason for this is that we locate needs at a deeper, more essential layer of existence than wants, preferences, and interests. We present the rationale and methodology of this framework more fully in our Theoretical Framework section below.

We ask questions of efficacy and extent:

- Efficacy - Did a UBI Plus intervention help participants meet their needs? If so, how?
- Extent - To what extent was this achieved?

We begin this article by introducing WorkFREE. We then present our theoretical framework, making the case for ‘UBI Plus’ - complementing a basic income with additional ‘pluses’, justifying our needs-based approach to design, implementation, and evaluation, and introducing Manfred Max-Neef’s (1991) ten fundamental human needs as our chosen framework for evaluation. Next, we specify the mixed-methods methodology used to collect and evaluate the quantitative and qualitative data presented in this article. We then present our findings for each of Max-Neef’s needs, presenting the ways and extent to which UBI Plus helped or did not help participants to meet their needs.

In our Discussion section, though we argue that we find compelling evidence for the significant efficacy of WorkFREE’s UBI Plus as developmental intervention based on a fruitful synergistic relation between its two components, we also emphasise the greater attention to gender difference needed for a more effective intervention. Finally, we make the case for advancing needs-based approaches to intervention design, implementation, and evaluation. We conclude that, though we do see potential for advancing UBI Plus as a ‘synergic satisfier’ (Max-Neef, 1991, 34) - an intervention able to help participants simultaneously meet not just material, but psychological and relational needs - we recognise the need for the model’s refinement in design, delivery, and evaluation.

## Introducing WorkFREE

### Project Overview

Operating between 2020 and 2024, WorkFREE combined development research with a pilot intervention in five urban informal settlements (*bastis*) in city name, India. WorkFREE was funded by X and managed by the University of X, the X, and a local NGO. Over 1,400 people living in 295 households in five discretely bounded settlements participated in WorkFREE. These are communities of lowest castes – Scheduled Caste (27%) and Other Backward Caste (71%) - arriving in city name over recent years and decades from rural villages; communities marred by high levels of widespread and entrenched poverty and indebtedness, alcohol and/or drug abuse, and misogyny and domestic violence. Whilst participants worked in many different activities, waste collection was the dominant occupation (42%) alongside domestic work (17%) and the preparation and sale of *rangoli* powder used to decorate the doorsteps of city homes (8%).

Three extensive household surveys were conducted over eighteen months to collect baseline (February 2022), midline (March 2023), and endline (November 2023) data. According to baseline data, almost 70% of participants were under 40 year olds (Table 1); 58% of participants had no formal schooling; and 93% identified as being Hindu whilst 5% identified as Christian. Almost half of participant households contained four or five individuals (Table 2) with almost 80% of households home to one or more children (Table 3). Only 3% of children were recorded as working.

**Table 1:** Participants’ ages

Age	Frequency	Percent
19–30 years	116	39.3
31–40 years	89	30.2
41–50 years	40	13.6
51–60 years	34	11.5
61–70 years	14	4.7
70 + years	2	0.7
Total	295	100

**Table 2:** Number of participants in households

B1.5 How many people live in this household (including you)	Frequency	Percent
1	29	10
2	27	9
3	55	19
4	74	25
5	72	24
6	22	8
7	14	5
8	1	0
9	1	0
Total	295	100

**Table 3:** Number of children in households

B1.6 How many children do you have? (living both in and outside of the household)	Frequency	Percent
0	63	21.4
1	47	15.9
2	85	28.8
3	77	26.1
4	17	5.8
5	6	2
Total	295	100

Pilot design eschewed randomized controlled trials in favour of a saturation approach in which all residents were invited to participate in the project.^2^ Bar a few individuals all invited consented to participate. Between May 2022 and October 2023, cash transfers were paid electronically each month for eighteen months to all adult individuals who received INR1000 (around $12). Mothers were given an additional INR500 for each child. This amount was informed by a proposal for a nation-wide basic income in India (Subramaniam 2017), calculated as a ‘top up’ to the World Bank Poverty Line. The average household received INR 2,722 ($32) per month, reflecting approximately 20% of its average income. The average household basic income in WorkFREE was around 11% of a living wage for a South Indian city (Medinaceli et al. 2024), representing a modest cash uplift to rather than replacement for employment income.

Between October 2022 and October 2024, the implementing NGO ran regular ‘Plus meetings’ in each participant communities – bimonthly until October 2023 and monthly thereafter.

### Project Implementation

**Fig. 1:**
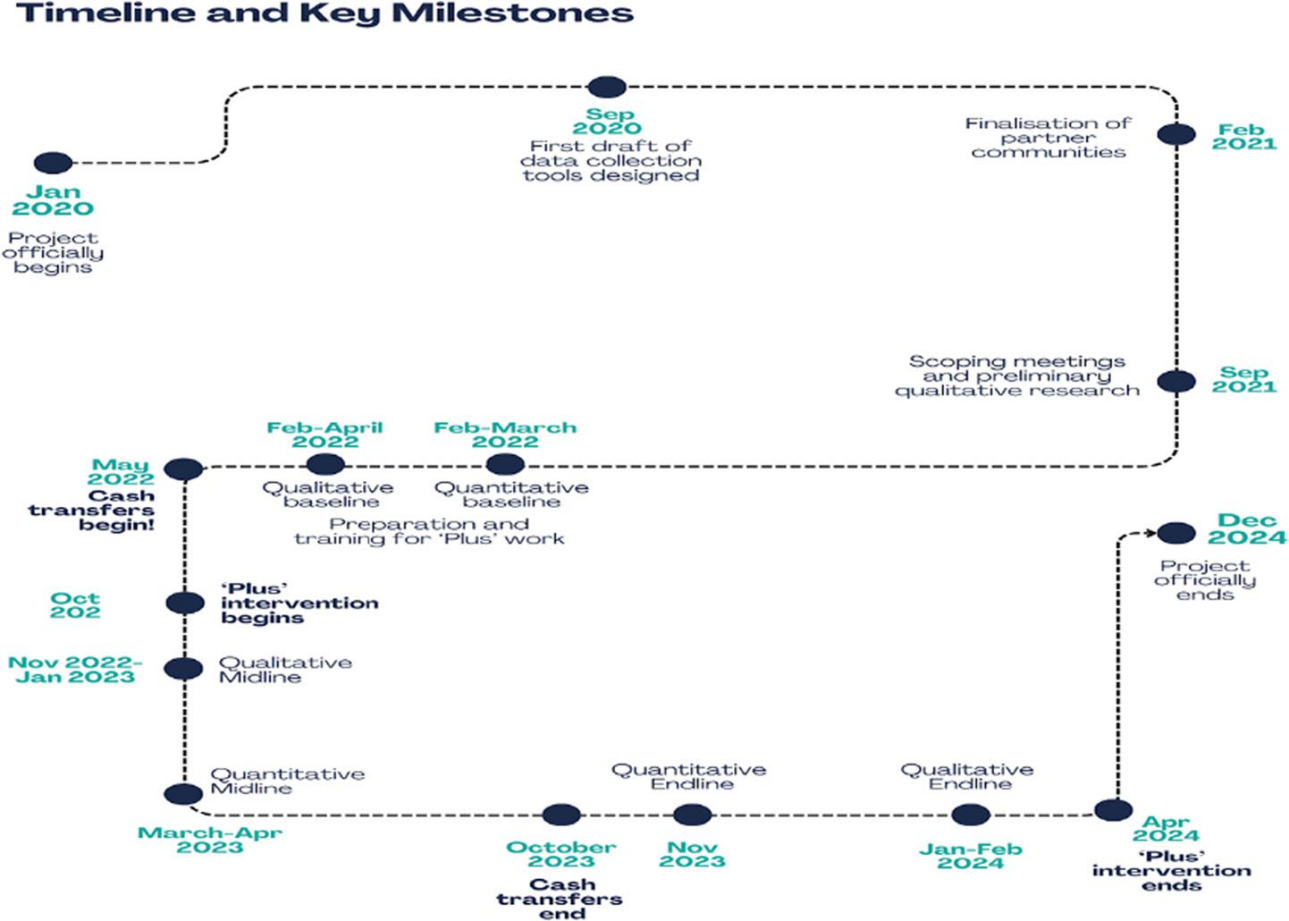
WorkFREE’s timeline and key milestones (Mathur et al., 2023)

The timeline of the UBI Plus intervention is presented in Fig. 1 below. Implementation began in earnest in February 2021 with visits to each *basti* to introduce the team and the project and to secure participants’ informed consent. The NGO team then supported all participants to ensure they were ready to receive electronic bank cash transfers with participants receiving their first cash transfer in May 2022.

The Plus component process began internally in March 2022 with a week-long series of workshops in city name facilitated by University of X project leaders. Used to working in more traditional directive rather than facilitative ways, the team participated in intensive, experiential initial and ongoing workshops that applied practical needs-centred frameworks; specifically, Nonviolent Communication (NVC) (Rosenberg, 1993), Human-Scale Development (Max-Neef, 1991), Kaufman’s (2020) Sailboat, Polyvagal Theory (Porges, 2017; Dana, 2018), Trauma-Informed Practice (Hübl, 2020), and Convergent Facilitation (Kashtan, 2021). Through the workshops, the facilitators communicated WorkFREE’s theory and practice of change, emphasising that the NGO team’s capacity to support participant communities to meet their needs would be determined by the extent to which the team could meet its own relational needs.

The ‘relational, needs-based’ approach to community organising that WorkFREE piloted differed greatly to the prior, more typical ‘problem-solving’ approach adopted by the partner NGO.^3^ Plus meetings were scheduled, regular gatherings focused on helping participants to explore their needs and generate and pursue potential ‘satisfiers’ of those needs (Max-Neef, 1991). Plus meetings were intentionally designed to cultivate deeper relational connection and to make visible and challenge entrenched practices of power. Their core features included:

- Being convened in public spaces;
- All participants and staff sitting on the floor in a circle so all could be seen and heard equally;
- The use of metaphors, drawings, and props to convey and generate ideas;
- The use of play, meditation, art, and active listening to cultivate safety, connection, and self-esteem.^4^

The original plan to hold monthly Plus meetings in each community for 18 months was scuppered by internal and external circumstances. Ultimately, meetings were held bi-monthly for the first twelve months and then monthly for the final six months.

Having presented an overview of WorkFREE, we now share our theoretical framework.

## Theoretical Framework

### Alternatives to GDP and Mainstream Social Policy

There is now a widespread critical rejection of a focus on material accumulation and economic expansion per se as either goal or indicator of societal development and human well-being (Daly, 1996; Hickel, 2019; Jackson, 2021). Moreover, in the context of ecological crisis, mere economic growth is incompatible with sustainability (Vogel & Hickel, 2023; Langridge, 2024). Whilst reconceptualisations of well-being and development centre on enhancing human capabilities (Sen, 1999), some theories focused on human need have found favour within the ‘growth-critical literature’ (Langridge et al., 2025, 1020; D’Alisa et al., 2015). Such theories eschew an earlier ‘basic need’ approach to emphasise the holistic nature of our needs as not just physiological, but psychological, emotional, and spiritual. Whilst how we pursue their satisfaction may be particular to time and place, such needs are consequently universal in nature (cf. Maslow, 1971; Max-Neef, 1991; Doyal & Gough, 1991; Ryan & Deci, 2017; Kaufman, 2020). Their holistic and universal nature make such understandings of human need relevant to reimagining institutions and policies for ‘sustainable welfare’ (Büchs et al., 2024).

One such policy alternative generating much discussion in this sphere is that of Unconditional Basic Income (Büchs, 2021; Fitzpatrick et al., 2022). Despite much interest, however, Langridge et al. (2025, 1021) have highlighted ‘a significant gap between such theoretical visions and the scope and focus of much of the empirical research’. In short, ‘there is a distinct lack of empirical research that considers UBI’s impact on human needs satisfaction’; ‘a major gap when considering UBI as a post-growth policy (ibid., 1024, 1021). This article builds on Langridge et al’s (2025) article - a needs-based evaluation of the HudsonUP basic income pilot - which, to the best of their and our knowledge, was the first to ‘empirically examine changes to holistic wellbeing under UBI conditions by employing a human needs approach (ibid., 1033).

### Advancing the Basic Income Agenda Through the WorkFREE Pilot

It was in the late 1980 s that criticisms of the inefficient, conditional, punitive, and stigmatising nature of European welfare regimes crystallised into an advocacy for a basic income that would acknowledge and satisfy the needs of all citizens not just for income security but human dignity (Van Parijs & van der Veen 1986). As a burgeoning number of pilot studies reveal the potential strengths and limitations of basic income’s contribution to advancing developmental goals and addressing social, economic, and ecological injustices, a small but growing number of scholars have begun to argue that ‘cash is not enough’ - that additional interventions should be integrated with cash transfers to maximise the developmental potential of cash transfers or to create a synergic interaction between the two (Roelen et al., 2017; Little et al., 2021; Howard et al., 2024; Mathur et al., 2025). In this context, we have seen an increasing number of pilot schemes complement the provision of unconditional cash transfers with what have come to be known as ‘pluses’ (Howard et al., 2024). One of the largest of such pilots was the WorkFREE project.

### Theory of Change

WorkFREE’s Theory of Change (ToC) understood development in terms of participants’ enhanced capacities to meet their personal and collective needs. We see human needs as lying beneath our wants, preferences, interests, or values. The experience of needing is essential not just to human beings, but all living organisms. Our needs are physiological, psychological, emotional, and spiritual (cf. Max-Neef, 1991; Maslow, 1971; Ryan & Deci, 2017; Kaufman, 2020). Thus, we understand well-being as the experience of need-satisfaction and disease as the experience of unmet needs. This understanding is informed by a substantial academic literature on well-being (cf. White 2015; Martela & Sheldon 2019; Sirgy 2021). The duration of time it takes to generate pathology may differ, but whilst violation of our need for Subsistence can soon threaten health and even life, violation of our need for Affection through, say, abuse or social isolation will generate not just psychological but even physical ill-health in young and old alike (Petitte et al., 2015; Carter et al. 2015). Such a holistic and realistic appreciation of the richness of our needs contrasts with a useful but ultimately reductionist and economistic definition of the ‘good life’ as the satisfaction of our supposedly *basic* (material) needs (cf. Hickel & Sullivan, 2024).

If we care about contributing to developmental changes that are effective and sustainable, a focus on all aspects of human needs and their satisfaction is vital. This ontological and ethical approach aligns with multiple recent contributions to this journal (cf. Ayala et al., 2025; Langridge et al., 2025; Paquet et al., 2025).

An appreciation of complexity theory informed our choice of relational community organizing as our Plus intervention over more predetermined and prescribed measures. An appreciation of the world as immeasurably complex leads to a recognition of change as unpredictable and emergent (Chesters, 2018; Boulton, 2024). We therefore sought to create conditions for positive change to emerge from participants’ interactions and imaginations (Burns, 2007).

Our Theory of Change embraced this holistic perspective on needs to acknowledge that human beings’ needs are both material *and* psychological and relational. In earlier articulations, we adopted a more simplistic attributional vision, anticipating a division of labour between cash transfers meeting participants’ material needs and our Plus community meetings helping participants to meet their psychological and relational needs. However, as our findings show and we explore in our discussion, our analysis gave way to a more sophisticated understanding of the synergistic relationship between cash and Plus.

###  Using Max-Neef’s Fundamental Human Needs as Evaluative Framework

Manfred Max-Neef (1991, 16, 24) makes an indispensable ‘fundamental’ distinction between needs and ‘satisfiers’ - ‘everything which, by virtue of representing forms of Being, Having, Doing, and Interacting, contributes to the actualization of human needs’. For Max-Neef (1991, 18) human needs are universal - ‘finite, few, and classifiable’ - whereas the ‘satisfiers’ available to us to deploy to meet those needs are historically, culturally, politically, and economically particular. Needs may be universal, but ‘the ways in which we experience our needs…are ultimately subjective’ (ibid, 26).

Max-Neef (1991, 8) specifies ten ‘fundamental human needs’:

1. **Subsistence** - As biological organisms, meeting our need for subsistence is first meeting our need for continued physical survival and adequate nutrition for longer-term flourishing.
2. **Safety (Protection)** - We prefer the word ‘Safety’ over Max-Neef’s original preference for ‘Protection’ because whilst Protection points to external intervention, Safety describes an internal experience. Consequently, using the word ‘Safety’ enables us, following neurobiologist Stephen Porges (2017) to define it primarily not as the removal of external actual or perceived threat, but as the attainment of internal psychological safety through the relaxation of the autonomic nervous system, usually through authentic connection with others.
3. **Affection** - We use the term ‘Affection’ here to capture a broad range of psychological needs from authentic, meaningful connection to care and love.
4. **Understanding -** ‘Understanding’ is another comprehensive term capturing our need to learn, to develop our capacities for making sense of our lives and our world, to understand self and others, as the first step to enacting effective change.
5. **Participation** - Our need to participate meaningfully in our community, our economy, and our society is a profound and well-documented need. Arnstein’s (1969) famous ladder of participation reveals a spectrum in the qualitative variation of participation; a variation significantly determined by relations of power.
6. **Idleness -** Though a word used pejoratively in our culture, the word ‘Idleness’ refers to our physical and psychological need for rest, respite, restoration, and recreation.
7. **Creation** - ‘Creation’ refers to the universal human need to be creative; to express our creativity in the infinite and unique ways open to us.
8. **Identity** - The need for ‘Identity’ relates to our need for a clear sense of self and, moreover, of self-esteem from within and of dignity and respect from others. Rejecting ontological individualism, the ‘self’ that we refer to here is not just the individual self but includes a collective sense of self that a community or even society may feel and cultivate.
9. **Freedom** - The need for ‘Freedom’ captures our need to speak, associate, and relate with others with no infringements and to experience and enact autonomy and agency in all aspects of our lives.

A tenth human need for **Transcendence**, was acknowledged by Max-Neef (1991, 27) but ultimately excluded from his list since he saw it as ‘not yet’ but ‘likely to become’ universal. Inspired by Abraham Maslow (1971) and Scott Barry Kaufman (2020, 6), we understand Transcendence as ‘awareness…expanded beyond the self’, being ‘motivated by higher values’, and having *‘*a deep sense of who [we are] and what [we are called] to contribute to the world’.^5^

Our decision to use Max-Neef’s framework for evaluating WorkFREE expresses neither our belief in the inferiority of other evaluative frameworks nor our uncritical acceptance but merely acknowledges how the framework manages to cover the range of human needs - from physiological to spiritual - with impressive clarity and brevity. However, it is absolutely vital to acknowledge that the isolation of particular needs for analysis and evaluation is a useful, but ultimately limiting exercise since, following Max-Neef (1991, 31) himself, our goal as researcher-practitioners engaged in a development praxis is to support the cultivation of *synergic satisfiers* that help individuals, households, and communities to meet *many needs simultaneously*. We are therefore ultimately interested in UBI Plus’s potential as a synergic satisfier.

Having introduced WorkFREE, justified our needs-based approach and our choice of Max-Neef’s framework for evaluation, we now describe our methodological approach to our research before sharing our findings.

## Methodology

The epistemological position that informed our methodological choices was one that eschewed positivistic causal claims in favour of a nuanced contributions analysis (Mayne, 2019; Apgar et al., 2020). We therefore sought to analyse our data to interrogate our initial Theory of Change, or concept of attribution of causal change – that the two components of our UBI Plus intervention, cash transfers and Plus meetings, would respectively meet participants’ material and psychological and relational needs.

As a mixed-methods research project, WorkFREE combined quantitative and qualitative research. Quantitative research took a pretest-posttest design (Dimitrov and Rumrill, 2003). This involved a large survey conducted at the baseline, midline, and endline of the project, administered to all households in the project (*n* = 295 across three rounds). Qualitative research was conducted primarily by two doctoral researchers and one postdoctoral researcher and took the form of ethnographic participant observation alongside themed Focus Group Discussions (FGDs) and semi-structured interviews with participants. Again, baseline, midline, and endline qualitative research was conducted between February and April 2022, November and January 2023, and January to February 2024. In total, over eighteen months of ethnographic and qualitative research was conducted.

In the production of this article, we have combined the processing, presentation, and analysis of all quantitative data (Sarath Davala, Santosh Malviya, Vibhor Mathur) with the thematic analysis of all qualitative data (Joel Lazarus, Vibhor Mathur, Neil Howard, Maria Franchi). Two researchers (Joel Lazarus, Nick Langridge) collaborated to choose the survey questions most relevant to each human need before corroborating this selection with the wider team.

To mitigate confirmation bias in data selection, transcripts were uploaded to Dedoose, a web-based tool for research analysis and thematic coding. For this research, qualitative data were collaboratively deductively coded according to Max-Neef’s ten fundamental human needs.

We are conducting research into a pilot that we ourselves oversaw the design and implementation of. We therefore inhabit insider positionalities in relation to WorkFREE with significant ethical and methodological consequences (Mathur, 2024). Our actions to address these have centred on practising an ongoing, shared reflexivity when taking collective decisions concerning matters of inclusion and exclusion, interpretation, and presentation of data.

## Findings

We now present both quantitative and qualitative evidence for the ways in and extent to which WorkFREE’s UBI Plus intervention enabled participants to meet each of Max-Neef’s ten fundamental human needs. In what follows, our quantitative data capture participants’ perceptions of their experience of the UBI Plus intervention whilst we use our qualitative data to ascribe attributions between the intervention and changes in individuals’ and communities’ lives. All data presented here is from our endline survey.

We first share data on the most important contributions of cash transfers to participants’ lives (see Table 4). The participants of 293 (of 295) households chose their three most popular options.

**Table 4:**
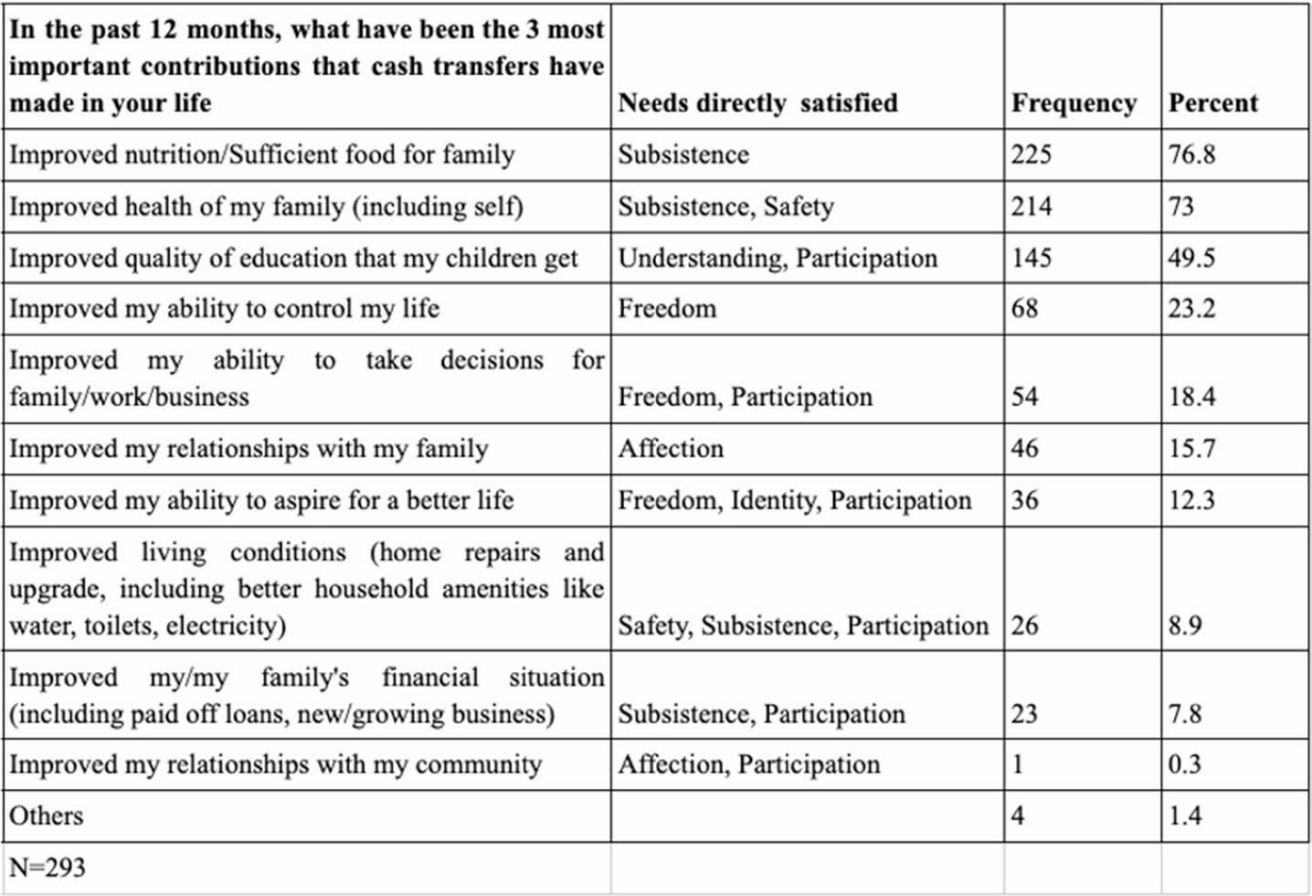
The most important contributions of cash transfers to participants’ lives

Table 4 shows how, while cash transfers contributed primarily to helping participants meet their physical needs for Subsistence and Safety, they also seem to have contributed to helping participants meet their broader psychological needs. That said, in our design and evaluation of UBI Plus, we saw cash transfers and community organising as a synergic whole and do not seek to disaggregate attribution of their respective impacts. Consequently, we will now present our findings regarding the contributions of the cash transfers and the ‘Plus’ community intervention together.

### Subsistence

Quotes conveying the daily struggle of so many participants merely to subsist were commonly expressed early on before or during the early months of the cash transfers. Two examples are:

We worked today, so we can eat today, sir. (WFP01 FGD)

My husband cannot go to any work. If we eat one meal, we are not sure about the next. Everyone here is the same. (WFP02 FGD)

We determined that just one household survey question was relevant to the issue of subsistence:

1. Was the household’s income in the past four weeks more than sufficient, about adequate or insufficient for your household’s food needs?

For question SUB1, we find a near-halving of reported food insufficiency from the rates reported in baseline and endline surveys (from 61.7% to 37.6%) and a concomitant near-doubling of reported sufficiency (from 31.6% to 60.3%). Whilst this is to be celebrated, it must be sadly acknowledged that over a third of respondents still reported household food insufficiency in the endline survey.

For very many participants and their households the impact of 18 months of cash transfers was significant. Many households simply ate more food:

The money that you provide us brings us more food to the table. Instead of twice a day, now we can have meals thrice a day. (WFP03 FGD)

Whilst the cash transfers helped many households eat, for many others they helped to: build a small buffer between them and crisis in the form of savings; to pay off existing or avoid taking on additional debts; and to meet their need for subsistence in the form of paying for healthcare costs. The following three quotes respectively convey these three ways of using the cash transfers:

Household saving - There was…no big financial issue at home at that point. So, I thought I could keep the money for the future and invest it in gold. But then my husband’s business dropped and so this money was a blessing. All the household needs were covered through this money. (WFP03 FGD).

Debt repayment/avoidance - We were happy that we don’t have to at least beg for anything or take any loan for emergencies. (WFP04 FGD)

Healthcare - It was definitely a significant thing. My grandson survived on this money. It was for injections and medicines. (WFP05 FGD)

Ethnographic observations and participant testimonies highlighted a reduction in the number of weekly visits from private loan agents in the communities (down from three days a week to one), and of participants uptake of such loans. In the endline survey, 37% households reported repaying long standing loans, of whom 52% attributed this ability to the intervention.

### Safety (Protection)

In the early days of meeting and speaking with WorkFREE participants expressions of feelings of unsafety like the following examples were often heard:

There are no things to say. We used to say but now no one [in the community] is trustworthy. (WFP06 FGD).

I request you to install a CCTV camera here, madam…I had a necklace and it was stolen. (WFP07 FGD)

We chose the following household survey questions to capture meaningful data on the satisfaction of the need for Safety:

1. Does the person have any medical insurance?
2. I feel safe in my house.
3. I feel safe in my community.
4. I feel safe at my workplace.

We include question SAFE1 about medical insurance to capture data concerning both the increased objective physical safety and an increased sense of psychological safety provided by (effective) medical insurance.

With regard to question SAFE1, we find a small overall increase from baseline to endline surveys in the percentage of respondents stating that they have medical insurance: from 6.3% to 11.2%. However, this rise is accounted for by a four-fold rise in respondents with government-provided insurance (from 2.1% to 8.8%), whereas the percentage of holders of private medical insurance actually fell from 4.2% to 2.4%.

As for questions SAFE2 to SAFE4, we find the following. The percentage of respondents strongly agreeing that they felt safe in their houses rose between baseline and endline surveys from 84.4% to 89.2% with the percentage of respondents disagreeing falling from 3.4% to 0.6%. The percentage of respondents strongly agreeing that they felt safe in their communities rose from 70.5% to 88.1% with the percentage of respondents disagreeing falling from 4.4% to 0.6%. Finally, the percentage of respondents strongly agreeing that they felt safe in their workplaces rose from 68.8% to 84.1% with the percentage of respondents disagreeing falling from 7.4% to 2% (see Table 5).

**Table 5:**
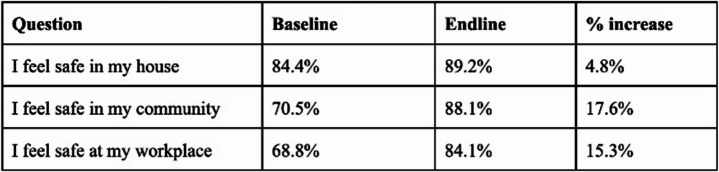
Percentage of respondents strongly agreeing to feelings of safety

Reports of such high levels of feelings of safety contradict the feelings of unsafety communicated by participants like those quoted above. We think this contradiction is best explained methodologically by a potentially problematic framing of the questions posed in our survey. That said, even accepting this flaw, the data still point to significant upward trends, nonetheless.

The qualitative evidence here points first to the *feeling* of safety engendered within participants when they realised that they really would receive a guaranteed additional and unconditional income each month for eighteen months. The guarantee of the cash transfers also helped participants feel safer through a mutual sense of enhanced creditworthiness:

It was a huge help. It was really helpful in taking care of the kids. If there was an emergency and I had to take the kids to the hospital, I just borrowed from others, promising them I would return the money the next morning by making a withdrawal from my bank account (UBI)…I had courage and security then. (WFP08 interview)

In community Plus meetings, facilitators used play, art, singing, and meditation alongside exercises for practising ‘active’ or ‘deep’ listening to cultivate safety and connection within the community groups. Implementers report seeing even initially hesitant participants feel increasingly ‘comfortable in their own skins’ and even ‘becoming like children again’ (WFI01 interview). A shared sense of safety was also cultivated by implementers themselves sharing personal stories in meetings. These acts of vulnerability were reciprocated by participants.

The safe space created by Plus meetings was particularly vital for female participants. In this space, women could at least begin to overcome the alienation of their individual suffering of male violence and share their stories, compassion, and solidarity (Lazarus et al., 2025).

These strong foundations of safety generated greater capacities for resolving conflicts in several participant communities. In one such community, for example, Plus meetings became a mechanism first for the community itself to heal relationships after the trauma of a sudden and forced eviction from their long-standing slum homes and removal to an apartment block on the very edge of the city. Later, with the support and facilitation of WorkFREE implementers, Plus meetings were used by that community to establish better relations with another community with whom it had been forced to live as neighbours (Lazarus et al., 2025). One resident of that community reflected thus:

We sit and talk and discuss many things. There is a lot of change among us with the help of meetings. Initially, we used to fight a lot but now our relationships have improved. We don’t fight like before. (WFP09 FGD)

### Affection

The following quotations reflect commonly articulated expressions of disunity, disconnection, and isolation within some participant communities in the early days of the WorkFREE intervention:

If there was unity in this basti society, things would have happened long ago. (WFP03 FGD)

Everyone is by themselves here, madam. They do their own work. It is just that no one asks for other’s help here. (WFP04 FGD)

Every individual is for themselves and they look after themselves. No one goes to the other house in need of something. (WFP05 FGD)

We identified the following questions from the household survey as most effectively capturing data concerning this need:

1. In the past 12 months, what would you say has been the impact of cash transfers on your relationship with other members of your household?
2. In the past 12 months, what would you say has been the impact of cash transfers on your relationship with other members of your family who do not live in your house in this basti?
3. I feel content/satisfied with my family relationships.
4. I often feel lonely.

Questions AFF1 and 2 were only asked as part of the endline survey. We see these two questions capturing data directly relating to perceptions of the quality of relationships within immediate and extended families. Further to this, question AFF3 essentially invites respondents to evaluate whether their need for Affection is being met within their families. Finally, Question AFF4 focuses on loneliness as the experience of the unmet need for Affection.

Regarding questions AFF1 and 2, remarkably 92.5% of respondents in the endline survey reported that cash transfers had improved their relationships with other household members whilst 91.1% said that cash transfers had improved their relationships with family members living outside of their houses. As for question AFF3, there was a slight increase of 4.7% in the number of participants reporting strong contentment with their family relationships (from 83.4% to 88.1%). Finally, regarding question AFF4, the number of respondents strongly agreeing that they often feel lonely fell from baseline to endline surveys from 36.9% to 24.1% whilst those disagreeing rose from 41.3% to 48.5%.

The greater sense of Affection - of connection and care - that the survey data convey was cultivated in Plus meetings. One implementer recalled how helping participants to ‘introspect and reflect on themselves’ and their ‘emotional needs’ enabled them to move beyond previous ‘ego clashes and jealous feelings’ and ‘start to understand one another more deeply’ (WFP02 FGD). As the intervention ended, female participants expressed their gratitude for being able to ‘share our sorrows’ and how ‘we all could really understand and relate to them’ (WFP10 FGD). Another male participant reflected how ‘by attending the meetings, I started to respect the elders before whom I never respected’ (WFP11 interview).

Unconditionality and universality of the intervention had a significant effect on positive social relations. Participants reported having ‘more in common’ and opportunities for ‘small talk’ with their neighbours about the intervention. We also witnessed the universality of cash promoting greater mutual aid and collaboration. This was witnessed in older participants being assisted by younger participants in generations in accessing digital and financial services. These findings contrast with reports of envy, distrust and conflict in targeted cash transfer projects where only some participants in a community receive the support (MacAuslan and Riemenschneider, 2011; Ellis, 2012; Grisolia et al., 2024).

This enhanced capacity to mutually meet the need of Affection was also an aspect of the relational journey between participants and implementers. As feelings of safety and affection grew between them, participants and implementers were able to move beyond playing the transactional roles of victims/saviours to become partners in a developmental process.^6^ The following quotations from participants are representative of sentiments increasingly expressed as the WorkFREE intervention unfolded:

We find you like our relatives.

We feel that you are our family members.

You can just take the money out of the equation completely and we still feel a lot of relation to you. (WFI03 FGD).

One implementer attributed this to the radical unconditionality that imbued the WorkFREE ethos and Theory of Change.

Cash transfers have stopped, but the fact that [participants] are still so keen on maintaining those relationships, I think, is a product of our emphasis on…unconditional love…rather than any sort of transactional or goal-oriented endeavour…The efficacy of the relationships that we have forged has sort of enabled, you know, a space of compassion, really, that I think can have been able to do a lot. (WFI04 interview)

### Understanding

We identified the following questions from the household survey as most relevant to capturing data relating to the need for Understanding:

1. Do your kids go to school?
  - In the past 12 months, did any of your children not previously going to school start going to school?
  - Was this change due partly or fully to the cash transfer?
2. I am able to learn new things.

The questions for UND1 concerning school attendance relate directly (albeit uncritically) to school and formal education as a satisfier of our need for Understanding and, secondarily, to the role of cash transfers in improving parents’ capacities to meet this need for their children. The question UND2 might relate equally to our need for self-esteem, captured here by the need for Identity.

Regarding question UND1, the percentage of respondents reporting that their children were attending school rose from 52.2% to 60.7% from baseline to endline surveys. 8.1% of endline survey respondents reported their child/ren newly attending school in the past twelve months. 91.7% of these respondents attributed this change partly (29.2%) or fully (69.5%) to their receipt of cash transfers. Finally, in response to question UND2, there was a 9.1% increase between baseline and endline surveys of respondents somewhat or strongly agreeing that they felt able to learn new things (from 81.7% to 90.8%), whilst the number of respondents disagreeing with this statement halved from 16.6% to 8.2%.

37.5% of endline survey respondents indicated that they had used their cash transfers to pay for education costs. Researchers heard many participants confirm this as the following quote from a male participant demonstrates:

Once, I was supposed to pay my kids’ school fees and ran short of money. The [cash transfer] was credited at that moment, and the fees were paid immediately. I felt relieved. (WFP13 FGD).

Many participants identified Plus meetings as places of learning and this as the reason for their continued participation:

Regardless of the money I am attending these meetings because I am learning new things which I don’t know and I feel happy about it, so that is why I keep attending. (WFP06 FGD)

In matters of confidence and self-respect, this project has been very helpful. I learnt many things, like how to present myself, my ideas, how to listen to others’ views… It’s about how we want to work together for the community. (WFP06 FGD)

In the previous section on the need for Affection, we showed how Plus meetings centred on processes of reflection and learning. Plus meeting participants also reported gaining a deeper and more critical understanding of their community:

We want meetings….we are getting the awareness, we are getting some wisdom, we came to know how to negotiate, we are learning how to behave properly. (WFP12 FGD)

This critical understanding empowered some to challenge certain aspects of what they perceived as unjust power structures (see the section on Participation below).

### Participation

We determined that the survey questions PART1 and PART2 best captured data relating to community and political participation. Question PART3 captures something relevant to power relations in participants’ communities.

1. Community participation.
  - Is anyone in the household associated with:
    i. a credit or savings group/committee/chit group/self-help group?
    ii. a social group or festival committee?
    iii. a caste association?
    iv. another club or association?
  - Does your neighbourhood have any neighbourhood welfare association/organisation?
2. Political participation:
  - Is anyone in the household associated with:
    i. an employee/workers association or trade union?
    ii. a political party?
  - Power:
  - In the past 12 months, in your opinion, what has been the impact of cash transfers on your ability to raise concerns/complaints to community leaders/public officials?

We found very small increases in the endline survey in the number of participants saying that someone in their household was associated with the various types of organisation listed in questions PART1 and PART2, suggesting minimal changes in community and political participation. However, we found that the number of people in the endline survey reporting the existence of a neighbourhood welfare association or committee more than doubled from the baseline survey from 7.5% to 18.6%. We also found that in answer to question PART3 in the endline survey, 64.2% of respondents reported an improved ability to voice concerns or complaints to community leaders versus 17.1% who noted no change. At the same time, only 4.8% of respondents reported an improved ability to voice concerns or complaints to public officials, suggesting that if there were a meaningful improvement in power relations it was limited to intra-community relations.

Plus meetings were based on an ethos of radical equality - ‘In community meetings everyone will get an equal chance to speak and share things; [something that] hasn’t happened in my life’ (WFP13 FGD). This allowed many participants, above all women, who had historically been ignored or overlooked to be recognised and to participate in their community (Lazarus et al., 2025). Though patriarchal relations of course remained entrenched, this experience did allow some men to reflect on and revise their perspective and practices. For example, one male leader of one participant community admitted that his participation in Plus meetings had made him realise that ‘everyone has something to say’ and therefore decide to ensure that in his own household everyone would be able to contribute to making decisions (WFP13 FGD).

Women participants reported that, through the Plus meetings, their increased capacity to meet their needs for Affection - empathy and solidarity - was a foundation for enabling them to overcome alienation and address issues collectively. In different communities, women came together to: establish mutual support groups for their small businesses, set up pooled savings funds, and demand the establishment of a council-run kindergarten in their community. Women who regularly participated in Plus meetings also became Executive Committee members of the city name Garbage Collectors’ Collective which was jointly established by the WorkFREE implementation NGO and WorkFREE participant communities (Lazarus et al., 2025). One woman saw the Plus meetings as creating an opportunity for women to show the men that ‘when we go to these meetings and when we go out of our homes to do something like that we can achieve things like these’ (WFP14 FGD).

It was through Plus meetings that women in certain participant communities came to air their grievances and even challenge the informal, often self-appointed leadership of various men. In one community, for example, during one meeting, the women voiced their belief that nothing would be achieved by lobbying the traditional leaders and decided to establish a self-help-style group and choose their own leaders without the men who they saw as routinely ignoring and sidelining them whilst achieving nothing.

### Idleness

Over half of all households in the WorkFREE study were working long hours all seven days with any time taken off from work penalised by a loss of income or even termination of employment. The following quote taken from an FGD succinctly summarises the position for so many participants:

Participant: We have to go [to work] every day, madam.

Interviewer: Why do you go every day?

Participant: If we do not go every day, then we won’t have food. (WFP04 FGD)

The following quotes reveal conditions in which participants felt wholly unable to meet their need for Idleness early on in the WorkFREE intervention:

Our problems are inescapable. This condition is never going to change. We have to keep working like this forever. (WFP12 FGD)

Everyone has their own work. There is no free time…Everyone is busy. And there is no time to talk to anyone. (WFP13 FGD)

We found only one survey question that meaningfully related to this need:

1. How many days in a week do they work for pay?

We chose this question based on the logic that a reduction in the days taken up by paid work would generally suggest an increased capacity to meet one’s need for Idleness.

We found an 8.6% fall from baseline to endline survey in the percentage of respondents working seven days a week from 55% to 46.4% with a corresponding 10.1% increase in the number of respondents working six days a week from 31.3% to 41.4%. The number of respondents working five days or less remained small and relatively unchanged.

As exemplified in the following moving quote, the receipt of regular cash transfers seemed to increase participants’ not just material but also psychological capacities to absorb the financial costs of taking time off from work. This finding aligns with other similar arguments and findings (Raventós, 2014; Langridge, 2024).

Some days I wake up and my entire body is aching. I just can’t get up. These days I tell myself, it’s okay. This money is coming. I can afford to not go to work. (WFP15 interview)

Beyond the capacity of cash transfers to help participants enhance their capacity to meet their need for Idleness in a limited but significant way, Plus meetings were at least a place of respite and ease. Again, female participants in particular expressed gratitude for a space to ‘just sit and talk’ and ‘escape from the responsibilities and problems’ of their lives:

Without any troubles, we could just sit and talk and listen to each other. We never did something like that, so we felt good. (WFP12 FGD)

Playing games and doing art were particularly appreciated in this regard.

This equalisation in the recognition if not the realisation of the need for Idleness was a radical expression of WorkFREE’s principle of unconditionality.

### Creation

We identified only one question from the household survey relevant to this need:

1. I am able to be creative in my life.

Remarkably, between the baseline and endline surveys there was a large increase of 30.2% in the number of respondents strongly agreeing to question CREATE1 (from 19.3% to 49.5%). The percentage of those who strongly disagreed remained constant (from 17.3% to 15.6%).

Researchers have shown how poverty inhibits our capacities for long-term planning and dampens our aspirations (Sen, 1979; Appadurai, 2004; Mullainathan and Shafir, 2013). When one experiences the world as a place of danger and scarcity one’s need for Creation is met only in the creative ways one finds to navigate danger and survive scarcity. We found that the combination of cash transfers and Plus meetings itself *created time and space* to enable participants to move beyond crisis management and expand their personal and collective imaginations of the possible. WorkFREE implementers ran meetings focused on envisioning alternative futures to support this process; a process that generated the kinds of actions listed earlier.

Ten people sit together and talk together…We talk to each other and get to know what the other person is dealing with and we can suggest to each other new ways to deal with the problems. (WFP07 FGD)

Furthermore, the combination of receiving cash transfers and being with other community members in regular meetings made ‘sitting and talking about’ their needs and future plans ‘worth it’:

Many people come and talk about how life can be improved. But it’s only when everyone feels like we can do something about it that it’s worth it. Otherwise we are just talking in the air. (WFP16 FGD)

A young widow who lived alone narrated how she ‘saw little purpose in having big hopes or plans’. However, WorkFREE implementers described how ‘over the course of participating in the Plus meetings, forming ties with members of her community and feeling empowered because of the cash she felt greater desire and purpose in her life’ (WPI05 FGD).

### Identity

The working experience of people living in participant slum communities is one of a daily violation of their need for respect and dignity. As the following quote demonstrates, this is particularly true for those who work as garbage collectors:

We go to households and collect from 600 households…In those 600 households, only ten people will call by our name, or ‘amma’ or ‘anna’ (sister or brother). The remaining people call us *khachra* (mule) or *chetthawalu* (garbage heap). They see us less. We are not getting respect. (WFP02 FGD)

We identified the following two questions from the household survey that we determined captured data relevant to the Identity need:

1. I am treated with respect and dignity at home.
2. I am treated with respect and dignity at work.

Both questions were added to the endline survey in response to requests from our team of qualitative researchers. Consequently, we have no baseline data for them.

90.5% of respondents strongly agreed with question ID1 that they were treated with respect and dignity at home. With what we know about levels of domestic violence in participant communities combined with the fact that surveys were predominantly conducted in participants’ own homes, this result suggests that many respondents may have been unwilling or unable to answer more freely. Why 93.3% of respondents either strongly agreed (83.1%) or somewhat agreed (10.2%) that they were treated with respect and dignity at work (ID2) when we know that a great majority of participants suffer disrespect and indignity we can only speculate relates to the questions’ wording.

The unconditional nature of both the cash transfers and the community work - in contrast to the usual conditional and targeted interventions and policies that participants were used to - was commonly mentioned appreciatively by participants. The offering of unconditional cash transfers alone was received by participants as an expression of recognition of their humanity, trustworthiness, and intelligence. Participants regularly conveyed the profound power of simply being seen, trusted, and valued:

Nobody trusts us. Nobody thinks ‘these people’ are good for anything. But this project trusts us. They think of us as real people and not just criminals (WFP17 interview).

These feelings of recognition were also articulated by those participants most acutely suffering from economic exclusion such as teenages, young adults, widows, and the elderly. One elderly woman, a former domestic worker who had to stop working due to ill health said:

Who am I? I am not a government employee. My kids don’t live here. My employers didn’t want me anymore. I’m just wasting away here to die. Then this money comes in. It makes me feel like I am also someone (WFP18 FGD).

This feeling of recognition was amplified by the unconditional approach of Plus meeting facilitators. In contrast to romanticised notions of poverty, prior to WorkFREE most participant communities enjoyed very low levels of community cohesion. Implementers were able to help participants meet their need for Identity simply by leading games in Plus meetings that helped participants to learn each other’s names.

Through games, we got to know ourselves and others in the community. We never knew that there were these many members in this community. These meetings helped us a lot. (WFP05FGD)

Through participating in Plus meetings, many participants reported an increased sense of personal and collective pride and self-esteem. One researcher who worked with focus groups of women from all participant communities noted a huge increase in confidence and capacities for self-expression alongside a general move toward a greater future orientation in their conversations.

### Freedom

A sense of a profound lack of freedom was routinely expressed by WorkFREE participants. Women, above all, constantly lamented how there was no choice when it came to marrying and staying with their husbands:

We can separate from our husbands and live alone happily if we have financial independence. In the present condition, we can’t ask our husband to stay with us or ask him to leave. We have no choice to say anything. Anything works only if we have money. (WFP11 interview)

We identified the following three questions from the household survey as capturing data relevant to this need:

1. I have the power to say no to uncomfortable situations and relationships.
2. I have freedom in deciding what work to do.
3. I am free to choose to live my life as I want.

Questions FREE1 and FREE2 convey something fundamental to the rationale behind the design of WorkFREE’s intervention - helping individuals and communities through cash transfers and community organising to attain or reclaim the ‘power to say no’ primarily to dangerous, undignified, or hyperexploitative work (Wilderquist, 2013), but, as question FREE1 expresses, also to other personal unwanted or abusive relationships. Question FREE3 captures a broader degree to which respondents feel free in their lives.

Regarding question FREE1, from baseline to endline surveys, we found a small 6.7% increase in the number of respondents strongly agreeing that they had the power to say no to uncomfortable situations and relationships (from 66.5% to 74.2%). Those disagreeing fell from 9.5% to 4%. For question FREE2, we found an increase of 10.5% in those strongly agreeing that they had freedom in deciding what work to do (from 75.3% to 85.8%) with those strongly disagreeing falling from 5.1% to 0.7%. Regarding question FREE3, we found an 11.9% increase in the percentage of respondents strongly agreeing that they felt free to choose how to live their lives (from 65.4% to 77.3%). Those disagreeing fell from 7.1% to 2.1%.

The freedom that came with the absence of conditions attached to the cash transfers - in contrast to most forms of state or NGO support - was greatly appreciated by participants. Women shared how the cash transfers had given them greater freedom from their husbands:

Participant 1: …. for everything, we were dependent on our husbands for small things ….

Participant 2: asking [rupees]100 would also be like, they used to ask us why do you want money? Even for [children’s] dresses also, we have to ask… Now, we can get our money; there is no need to ask for money from them for small things. (WFP19, WFP20 FGD)

Women also reported how this greater freedom empowered them to loosen relations of dependency and renegotiate relationships within their households:

With this amount, I can purchase tablets and some extra medication and get some good food. I don’t need to depend on my grandsons for money; sometimes I can even give the amount in return. (WFP21 interview)

Every month my brother had to come and top up my ration, or leave some money for medicines. These days I tell him its okay. I’m managing. (WFP22 interview)

Unfortunately, the cash transfers proved insufficient in this aspect. Whilst some women felt that the cash transfers had empowered them to demand and achieve more equity in sharing household responsibilities, none felt that the WorkFREE intervention had enabled a financial or cultural shift large enough to empower women to leave abusive husbands.

### Transcendence

We found one relevant question in our household survey:

1. I feel part of a group bigger than me.

Whilst implementers or participants might be able to point to moments of transcendence that occurred during Plus meetings, we as researchers cannot offer anything scientifically rigorous about the WorkFREE intervention’s capacity to meet this need. That said, interestingly, we found that the percentage of respondents strongly agreeing with this statement in TRANS1 almost doubled from the baseline reading of 36.9% to 72.5% for the endline result. At the same time, the percentage of those disagreeing more than halved from 11.8% to 5.1%. What we believe these responses point to are the enhanced levels and depths of social cohesion, co-operation, and mutual aid achieved through participation in the intervention.

## Discussion

In this article, we are analysing UBI Plus as a developmental intervention by conducting a needs-based evaluation of WorkFREE, a major UBI Plus pilot intervention. Specifically, we sought to evaluate three aspects of UBI Plus: efficacy - whether and how UBI Plus can help participants meet their needs; extent - to what extent; and synergy – what we can say about the synergistic relationship (or otherwise) between the two components of UBI Plus – cash transfers and community organising. Additionally, we emphasise the crucial importance of building acknowledgement of difference – in our case, concerning gender difference – into all stages of intervention design, delivery, and evaluation. Finally, we reflect on the utility of needs-based approaches to intervention design, implementation, and evaluation.

### Efficacy and Extent: UBI Plus as Synergic Satisfier

Our initial Theory of Change specified a simplistic division of labour between cash transfers meeting participants’ material needs and the Plus meetings helping participants to meet their psychological and relational needs. We now recognise a far more synergistic relation between the two components.

We can understand the synergic properties of UBI Plus in two ways. First, we can understand the intervention as a whole as constituting a synergic satisfier in the way that it helped participants to meet multiple material, psychological, and relational needs. Second, we can recognise how combining unconditional cash transfers with unconditional community organising itself creates a powerful synergy (Roelen et al., 2017; Howard et al., 2024; Lazarus et al., 2025).

With regard to efficacy and extent, the combination of quantitative and qualitative evidence offers compelling evidence for UBI Plus helping many participants meet not just their physiological, but their psychological and emotional needs to a significant extent.

Table 1 reveals how, unsurprisingly, participants were able to use cash transfers primarily to better meet their most basic need for Subsistence. However, even the data presented in Table 1 point to the *synergic* nature of cash transfers as a satisfier; a nature more clearly revealed by qualitative evidence showing how cash transfers contributed to helping participants meet needs such as Safety, Identity, Freedom, and Participation. Similarly, whilst qualitative data provide unsurprising evidence for how Plus meetings helped participants to meet psychological and relational needs, they also point to how participants used meetings to collaboratively pursue new satisfiers to meet their needs more effectively. Thus, our findings suggest the potential of UBI Plus as a synergic satisfier, with the *combination* of unconditional cash and community organising helping participants to meet more basic material and psychological needs, thereby establishing a robust platform for effective personal and collective action aimed at identifying and pursuing more effective and sustainable satisfiers.^7^

These findings build on those from the first article to offer a needs-based evaluation of a basic income pilot – Langridge et al’s (2025) evaluation of the HudsonUP pilot; a pilot that offered cash transfers only. Also using Max-Neef’s framework, Langridge et al. (2025, 1035) found that ‘needs satisfaction increased over the course of the intervention, albeit at a gradual pace’ and that ‘the intervention widened participants’ view of wellbeing’. However, they point to ‘the limited options available in the wider economy which remain unaffected by the provision of a UBI’ (ibid., 1035). To use an analogy taken from neo-liberal Washington Consensus era debates concerning economic policy in so-called ‘developing countries’ (Rodrik, 2008), Langridge et al’s findings suggest that it is not enough to ‘get the prices right’, i.e. to provide cash alone; it is equally vital to ‘get the institutions right’ – in this case, to support communities to come together to maximise the potential personal *and collective* transformative effects of the unconditional intervention. Whilst the example of the city name Garbage Collectors Collective is encouraging, more research needs to be conducted to explore the synergistic potential of UBI Plus to support communities to come together to resist and influence local bureaucratic and political institutions or even to form alternatives.

### Gender (and other) Differences in Efficacy, Extent, and Sustainability

All structural factors of injustice and oppression are more extreme for women in these participant communities and, of course, across India, Asia, and our world. To this dismal list we must add the severe conditions of patriarchy under which female participants live. It is through this lens that we must analyse the particular experience of UBI Plus for women. Whilst the cash transfers ostensibly gave women more freedom within their households, it seems that they were mostly ‘free’ to spend their money on household necessities rather than on more leisurely pursuits (often tobacco and alcohol) enjoyed by men. However, at the same time, the Plus meetings proved invaluable for women as a space for: safety through connection and care (Safety, Affection); leisure time outside of their households (Idleness); learning new skills and confidence for speaking out within and beyond their communities (Understanding, Freedom, Participation); and creating solidarity and community (Affection, Creation, Transcendence). We can never assume any gender positive outcomes from the provision of either a basic income or UBI Plus, but must instead systematically integrate Feminist pro-actively gender-sensitive frameworks into intervention design, implementation, and evaluation (Franchi, 2024). The same sensitivity must be adopted with regard to all other marginalized and oppressed groups.

### The Utility of Needs-based Approaches to Intervention Design, Implementation, and Evaluation

We hope and believe that this article demonstrates the utility of adopting a needs-based approach to interventions; from design to implementation to evaluation - particularly in embracing an holistic appreciation of human needs. Beyond the obvious continuity and transparency in maintaining the same framework throughout, our findings illustrate the power of evaluating interventions through clear, practicable needs frameworks like Max-Neef’s Human-Scale Development framework. The combination of quantitative and qualitative data - taken from both interviews and FGDs and quotations from Plus meetings - allow for insightful analysis. Again, we emphasise the essential nature of needing in this context leading to the generation of more accurate or meaningful data. The participant quotations cited in this article - a handful of many dozens collected through interviews - convey the ultimate indivisibility of our material and psychological needs and therefore the necessity for development to be conceptualised and practised in holistic or synergic ways, as recent authors in this journal have emphasised (Ayala et al., 2025; Langridge et al., 2025; Paquet et al., 2025).

## Conclusion

In conclusion, we find compelling evidence for the potential of UBI Plus as a developmental synergic satisfier. We also, however, acknowledge limitations in our model design and implementation; limits we explore more fully elsewhere (Lazarus et al., 2025). One such limitation was an insufficient consideration of sociological difference, particularly gender difference. We also acknowledge a need to develop a more systematic approach to monitoring and evaluation. Nonetheless, we do find enough evidence to suggest that such refinements in the design, delivery, and evaluation of UBI Plus would be worthwhile and look forward to working with others to advance unconditional approaches to ‘cash plus’ interventions within not just the basic income community but across wider development and welfare policy-making circles.

We will have more to say about the sustainability of changes in future publications after our post-pilot household survey data is analysed. Our current evidence points to differences across participant communities. Whilst in some communities there was skepticism that community meetings or other activities would continue, in others there were commitments that they would. Regarding the larger-scale innovations, above all the establishment of the city name Garbage Collectors’ Collective, there are encouraging signs that many are enduring.

Ultimately, the communities participating in WorkFREE experienced lives shaped by very poor and highly precarious housing, highly exploitative and undignified working conditions, and social and political marginalisation. We must be realistic about how sustainable the effects of one pilot could ever be. Nonetheless, there is enough evidence here to ask what might be if interventions like this could itself be sustained for a far longer period of time.

## Appendix 1

**Table 6:** List of anonymized interviewees with their reference codes

Respondent code	Respondent role	Respondent gender	Source of quotation
WFP01	WorkFREE participant	Female	Focus Group Discussion
WFP02	WorkFREE participant	Female	Focus Group Discussion
WFP03	WorkFREE participant	Female	Focus Group Discussion
WFP04	WorkFREE participant	Female	Focus Group Discussion
WFP05	WorkFREE participant	Female	Focus Group Discussion
WFP06	WorkFREE participant	Female	Focus Group Discussion
WFP07	WorkFREE participant	Female	Focus Group Discussion
WFP08	WorkFREE participant	Female	Interview
WPI01	WorkFREE implementer	Male	Interview
WFP09	WorkFREE participant	Female	Focus Group Discussion
WFI02	WorkFREE implementer	Male	Interview
WFP10	WorkFREE participant	Male	Focus Group Discussion
WFI03	WorkFREE implementer	Male	Focus Group Discussion
WFI04	WorkFREE implementer	Male	Interview
WFP11	WorkFREE participant	Male	Interview
WFP12	WorkFREE participant	Female	Focus Group Discussion
WFP13	WorkFREE participant	Male	Focus Group Discussion
WFP14	WorkFREE participant	Female	Focus Group Discussion
WFP15	WorkFREE participant	Male	Interview
WFP16	WorkFREE participant	Male	Focus Group Discussion
WFI05	WorkFREE implementer	Female	Focus Group Discussion
WFP17	WorkFREE participant	Male	Focus Group Discussion
WFP18	WorkFREE participant	Female	Focus Group Discussion
WFP19	WorkFREE participant	Female	Focus Group Discussion
WFP20	WorkFREE participant	Female	Focus Group Discussion
WFP21	WorkFREE participant	Female	Interview
WFP22	WorkFREE participant	Female	Interview

## Data Availability

The findings presented in this article are derived from quantitative and qualitative data generated from the WorkFREE research project. The data were generated by researchers at the University of Bath and India Network for Basic Income, both in partnership with a local NGO and over 1,400 people living in urban slums in city name, India. In accordance with national laws and university and grant donor regulations, all data have been anonymised and are stored securely by host universities. Consequently, these data are not freely available to be accessed and used. However, access to these anonymised data may be granted on request.

## Footnotes

1 See https://basicincome.org/about-basic-income/.

2 For information about the ethical and practical justifications for this approach see Mathur et al. (2023) and Howard et al. (2024).

3 For details on this contrast and the tensions and opportunities it generated see Lazarus et al. (2025).

4 For an in-depth practical presentation of this ‘relational, needs-based’ approach see Lazarus et al. (2026 forthcoming).

5 As such we recognise Transcendence not only as our highest or deepest universal and transhistorical need, but as the urgent meeting of this need as metaphysically crucial for humanity to ‘transcend’ the dualistic physicalist, positivist, and reductionist roots of our crisis (Lazarus, 2026) . However, evidence for WorkFREE meeting this need is not conducive to the approach we take in this article. Instead, for an analysis of the meeting of the need for transcendence in both this and two sister projects, see Lazarus (2026).

6 See Lazarus et al. (2025) for details of this journey ‘from saviour to partner’.

7 We have produced three articles that offer detailed and comprehensive presentations and analyses of our findings concerning the impact of our Plus meetings. Two of these articles (Lazarus et al., 2025; Lazarus, 2026) focus on the experience and perspectives of participants. A third article (Lazarus & Howard, 2026 forthcoming) focuses on the experience and perspectives of the pilot’s implementers.
